# Preliminary Cost-Effectiveness of Re-Purposing β-Blockers as an Adjunct Treatment for Women with Triple-Negative Breast Cancer

**DOI:** 10.3390/healthcare13222929

**Published:** 2025-11-15

**Authors:** Melanie Lloyd, Erica K. Sloan, Clara Marquina, Janet Bouttell, Omar Hassanien, Edoardo Botteri, Zanfina Ademi

**Affiliations:** 1 Health Economics and Policy Evaluation Group, Centre for Medicine Use and Safety, Monash University, Parkville 3052, Australia; 2 Monash Institute of Pharmaceutical Sciences, Drug Discovery Biology, Monash University, Parkville 3052, Australia; 3 Nottingham University Hospitals NHS Trust, Nottingham NG5 1PB, UK; 4 Department of Research, Cancer Registry of Norway, 0379 Oslo, Norway

**Keywords:** breast cancer, drug repurposing, health technology assessment

## Abstract

**Highlights:**

**What are the main findings?**

- Observational studies suggest that β-blockers, a low-cost generic medication, improve survival in triple-negative breast cancer.
- This economic evaluation estimates that adjunct use of β-blockers alongside standard care reduces healthcare costs and increases quality-adjusted life years when compared to standard care alone.

**What is the implication of the main finding?**

- While further monitoring of long-term mortality outcomes and adverse events is warranted, further tightly controlled randomised clinical trials will be difficult to justify economically given the extremely low cost and robust safety profile of generic β-blocker medications.

**Abstract:**

**Background/Objectives:** To evaluate the cost-effectiveness of β-blocker use in addition to standard care compared to standard care alone for women with triple-negative breast cancer (TNBC), with effectiveness measured by years of life lived (YLL), quality-adjusted life years (QALYs), and equal-value life years (evLYs) gained. **Methods:** A population cohort Markov model was developed to compare clinical and economic outcomes for TNBC treated with 1) lifelong β-blocker prescription initiated at diagnosis in addition to standard care versus 2) standard care alone. Life-table modelling was used to capture mortality over a lifetime horizon for the estimated eligible population of Australian women diagnosed with TNBC in 2022 (n = 767). Costs were derived from Australian healthcare perspective, and measured in Australian dollars (AUD) at 2022 prices with 5 percent annual discounting and AUD 28,000 willingness to pay threshold applied. **Results:** The model estimated 628 (95% CI 139, 1035) YLL, 526 (116, 865) QALYs, and 566 (125, 932) evLYs gained in the β-blocker group compared to standard care. The difference in health costs between β-blocker and standard care groups was AUD −935,116 (−2,365,417, 405,350). The β-blocker intervention was dominant over standard care in terms of both QALYs and evLYs gained. **Conclusions:** Preliminary modelling suggests that implementing β-blockers as an adjunct pharmacotherapy in the treatment of TNBC was more effective and less costly than current standard care. Further monitoring of long-term outcomes is recommended to validate the findings of observational and preclinical studies, and define the incidence, severity, and cost of β-blocker associated adverse events in cancer populations.

## 1. Introduction

Breast cancer (BC) is a leading cause of death in Australian women, accounting for eleven percent of deaths in the 45–64 years age group in 2021 [1]. The triple-negative molecular subtype accounts for approximately fifteen percent of BCs, and has a particularly poor prognosis due to the proliferative and aggressive characteristics of these tumours, which increase the likelihood of local and distant recurrence [2,3]. In triple-negative breast cancer (TNBC), tumour cells lack estrogen and progesterone receptors and do not amplify the HER2 protein, and therefore lack effective targeted treatments [4]. Current treatment of TNBC relies on chemotherapy, with recent approvals of immunotherapy combinations for early-stage and advanced disease, and sacituzumab govitecan for advanced disease [5,6,7].

Recent preclinical and clinical evidence has suggested that β-blocker drugs—medications developed and prescribed for the management of cardiac disease and hypertension—may be a potential adjunct pharmacotherapy in cancer treatment [8,9]. Processes that drive tumour progression are increased by β-adrenergic signalling [10,11,12]. β-blocker drugs inhibit these processes by blocking β-adrenergic receptors, which reduces cancer progression in preclinical models, both alone and in combination with standard treatments for TNBC [13,14,15]. A recent population-based cohort study and meta-analysis of observational data found that β-blocker use at diagnosis was strongly associated with improved BC-specific survival for the TNBC molecular subtype [16]. While randomized clinical trials are yet to evaluate the effect of β-blockers on TNBC recurrence or survival, phase II clinical trials in breast cancer patients show that β-blockers modulate biomarkers of metastasis and may be safely combined with neoadjuvant chemotherapy, suggesting that repurposing of this safe and commonly prescribed medication may improve outcomes in this aggressive disease [17,18,19].

As with all novel or repurposed health interventions, economic evaluation is desirable prior to implementation into routine clinical care to facilitate efficient allocation of finite health funding [20]. Early health technology assessment (HTA) has emerged as a tool to guide and inform decisions on product development and research prioritization [21]. While formal guidelines for early HTA do not yet exist, a recent Delphi study established economic evaluation as an appropriate methodology in early HTA [21]. In this context, health economic modelling can draw on preclinical or observational data from the literature, and set appropriate assumptions and scenarios, to apply simple exploratory models evaluating structural uncertainty. β-blockers are an older medicine with multiple affordable generic options available, and repurposing therefore avoids many of the drug development costs incurred for novel interventions. Propranolol, the β-blocker used in earlier phase II clinical trials for breast cancer, is available in Australia for an unsubsidized price of around only AUD 100 (EUR 56) per year. Early cost-effectiveness analysis is therefore important to enable policy makers to weigh up the research costs associated with further evaluation of efficacy in randomized clinical trials, versus the population level costs of funding the intervention for rare diseases such as TNBC [22,23]. We aimed to evaluate the cost-effectiveness of lifelong β-blocker use for women with TNBC in addition to standard care compared to standard care alone, with effectiveness measured by years of life lived (YLL) and QALYs gained. Cost-effectiveness according to disease stage at diagnosis (localized, regional, or distant—as defined in the aforementioned meta-analysis) was also explored [16].

## 2. Materials and Methods

A simple exploratory population cohort Markov model with one-year cycles was developed to compare clinical and economic outcomes for TNBC treated with the following approaches: (1) lifelong β-blocker prescription initiated at diagnosis alongside standard care versus (2) standard care alone. Life-table modelling was used to capture morbidity and mortality over a lifetime horizon from the healthcare perspective. Data were not available to explore the impact of TNBC on productivity to enable analysis from the societal perspective. No pre-specified health economic analysis plan was prepared.

Participants enter the Markov process at diagnosis in the ‘Alive with TNBC’ health state, and then remain in this health state or transition to the ‘TNBC-death’ or ‘Non-TNBC-death’ health states during each subsequent cycle depending of age-specific transition probabilities (Supplementary Figure S1). The primary outcome was incremental cost-effectiveness ratio (ICER) measured as cost per QALY gained. Costs were presented in Australian dollars (AUD); on 29 August 2025, AUD 1 = EUR 0.56. A discounting rate of five percent was applied as per Australian guidelines; prices are AUD (2022) and a willingness to pay (WTP) threshold of AUD 28,000 was adopted [24].

### 2.1. Population of Interest

Australian women aged 50–79 years diagnosed with TNBC were included in the analysis. Women under the age of fifty years were excluded as available evidence on the potential therapeutic effect of β-blockers in TNBC mainly derives from studies conducted in post-menopausal women or women above the age of fifty [16]. Women over the age of seventy-nine were excluded as aggressive diagnostic and treatment procedures are less likely to be pursued in women of advanced age due to significantly reduced likelihood of longevity and quality of life gains [25]. Women already receiving β-blocker treatment for another indication (estimated at 14.8 percent of women with TNBC [16]), and 20 percent assumed to have a contraindication to β-blocker use, were also excluded from the analysis (Supplementary Table S1). This assumption was deliberately conservative, and exceeded the 10 percent of the population with an absolute contraindication identified in a large registry-based study of β-blocker prescription in acute coronary syndrome [26].

The age-specific incidence of women diagnosed with breast cancer in 2022 was obtained from the Australian Institute of Health and Welfare [27]. The age-specific prevalence of the triple-negative molecular subtype among those diagnosed with breast cancer was obtained from the analysis of all breast cancer patients diagnosed in Norway between 2004 and 2018 performed at the Cancer Registry of Norway, as age-specific Australian data were not available. However, living standards and quality of publicly funded healthcare are comparable for Australia and Norway, and population level prevalence of the TNBC molecular subtype obtained from screening programmes is similar [28]. Women were grouped into age categories (50–54, 55–59, 60–64, 65–69, 70–74, and 75–79 years) and followed until death or when they reached 85 years of age (the average female life expectancy in Australia). Each age group was modelled separately to allow for age-related differences in the number of women diagnosed with breast cancer, prevalence of the triple-negative molecular subtype, health utility, and all-cause mortality (Supplementary Tables S1–S3). The total projected Australian female population by year of age for 2022 was obtained from the Australian Bureau of Statistics [29]. After applying the eligibility criteria described above, a population cohort of n = 767 women were entered into the Markov lifetable model.

### 2.2. β-Blocker Intervention

In the base case, a 40 mg dose twice daily of propranolol was prescribed, commencing at TNBC diagnosis and continuing for life. This dose has been used in previous phase II biomarker studies of propranolol in cancer patients, and is expected to achieve drug serum concentrations that have been shown in mice to slow cancer progression [17,18,30]. Life-long treatment is indicated as the risk of recurrence persists over the lifespan. As there are likely to be practical challenges associated with life-long adherence, this assumption was a key parameter explored in sensitivity analyses. Under usual care, it was assumed that no β-blocker was taken, given that women prescribed β-blocker medication for an alternative indication were excluded from the analysis.

### 2.3. Mortality Risk Under Usual Care

Annual risk of death was age-specific. The all-cause mortality incidence was taken from the Australian Institute of Health and Welfare General Record of Incidence of Mortality (Supplementary Table S2) [31], and assigned as the probability of a “Non-TNBC” death at each year of age. The probability of a TNBC death was fixed and was derived from the Cancer Registry of Norway five-year survival probability using the formula [annual death rate (per person year) = (−log(1 − α))/5], where α equals the five-year incidence of death (Table 1). The one-year rate was then converted back to a transition probability using the formula [annual probability of death = 1 − exp − r], where r is the one-year rate. Half-cycle correction was applied to years of life spent in each health state.

### 2.4. Intervention Efficacy

The impact of β-blocker use on TNBC-specific survival (overall and according to disease stage at diagnosis) was taken from a cohort analysis of over 2000 Norwegian women diagnosed with primary invasive TNBC between 2008 and 2018 (Table 1; hazard ratio for TNBC death with any β-blocker use: 0.66 (95% CI: 0.47–0.91)) [16]. That study further pooled the Norwegian cohort data within a meta-analysis of four previously published studies [16], the results of which were applied in a scenario analysis discussed below.

### 2.5. Health Utilities

QALYs adjust years of life lived by a health utility weight. Traditional methodologies assign a utility weight to each health state in the model, with utility values typically decreasing with severity of disease. A criticism of this approach is that QALY gains are much lower for interventions that extend the life of individuals with severe chronic illness, as is the case with most cancer treatments. To address this, an alternative approach was recently proposed whereby extensions to life gained through an intervention are valued consistent with the population-based utility average to derive “equal-value life years” (evLY) gained [38]. The evLY metric treats each life year gained equally, regardless of the underlying health state severity, which is considered a more equitable approach for diseases that significantly impact wellbeing. We therefore applied both the standard QALY methodology and the evLY approach in our model and present results from both.

Age group-specific health utility decrements for the “Alive with TNBC” health state were obtained from a large US-based observational study of BC survivors [33], and subtracted from age-specific population norms obtained from an Australian cross-sectional study of ~3000 individuals (Supplementary Table S3) [32]. In the year of TNBC-related death, an individual was assigned a terminal phase utility decrement of 0.11, consistent with the findings of a recent systematic review and meta-analysis [34]. A population-based utility average for Australian females of 0.90 was assigned for the evLY analysis [32].

### 2.6. Health Costs

The annual cost for daily dosing with propranolol was taken from the Pharmaceutical Benefits Scheme (PBS) (Item 2566C; AUD 113.95) [35]. The annual health cost for management of BC under standard care was taken from an Australian study that evaluated health costs for cancer care [36]. Administrative healthcare data was used to assign a top–down population level average cost for cancer management by tumour type and years since diagnosis, rather than disease stage. Annual BC treatment cost was highest in the first year after diagnosis (AUD 42,409), with declining annual costs assigned out to 5 years post-diagnosis (Table 1). A cost of AUD 44,327 was assigned in the terminal year for each TNBC-related death [36]. Non-TNBC-related deaths were assigned an average cost of AUD 5844, consistent with previously published economic evaluations of chronic disease in Australia [37]. All published cost parameters were inflated to 2022 prices using the Australian health price index [39].

### 2.7. Sub-Group Analysis

Cost-effectiveness was compared for each age group cohort (as defined above). A sub-group analysis was also conducted to compare the cost-effectiveness of β-blocker use according to disease stage at diagnosis. The proportion of the population assigned to each disease stage (60.5 percent localized, 35.6 percent regional, and 3.9 percent distant) was obtained from the Cancer Registry of Norway. Baseline TNBC-specific survival and treatment effect were varied according to disease stage (Table 1). Annual treatment costs were not varied according to disease stage.

### 2.8. Scenario Analysis

A number of scenarios were undertaken to explore parameter assumptions. Scenarios one and two varied the discount rate to 0 percent and 3.5 percent, respectively, as recommended in the Australian Pharmaceutical Benefits Advisory Committee submission guidelines [20]. Scenario three applied a smaller TNBC-specific mortality effect size for β-blocker treatment plus standard care versus standard care alone (RR = 0.74 compared to 0.66 in the base case) taken from a published meta-analysis [16]. As the optimal drug and dose for β-blocker treatment of TNBC is unknown, scenario four evaluated an alternative β-blocker drug, carvedilol, at 12.5 mg twice daily (PBS item code 8257N; annual cost AUD 258.18 per person) [35]. Carvedilol is used for the treatment of cardiac complications associated with chemotherapy use in cancer patients [40,41]. Scenario five retained propranolol as the drug choice, as in the base case, but increased the dose to 80 mg twice daily (annual cost AUD 227.90), the maximum dose used in biomarker studies of propranolol in BC [17,18]. Finally, in scenario six, the annual BC treatment cost was extrapolated beyond year five (for life) at AUD 2838 per annum until the terminal year.

### 2.9. Sensitivity Analysis

Deterministic sensitivity analysis was undertaken to explore uncertainty around the incidence of TNBC, TNBC mortality rate, effect of intervention, health utilities, and costs. Upper and lower parameter ranges were 95 percent CI where published, and the standard error was assumed to be 10 percent of the mean for costs (Table 1). Probabilistic sensitivity analysis (PSA) was performed for the base case and disease stage sub-groups (using 1000 iterations) to account for uncertainty in multiple parameter (effect of intervention, health utilities, and costs) sampling from the distributions listed above and summarized in Table 1. The mathematical logic and internal validity of the model was tested (Appendix A). 

### 2.10. Value of Information Analysis Methods

A value of information analysis was conducted with data generated from the probabilistic sensitivity analysis to estimate the cost of uncertainty in the model and the value of future research to reduce this uncertainty. A non-parametric approach was taken to determine the population expected value of perfect information, which describes the difference between the expected net-benefit with current versus perfect information [42]. Here, we assumed that lifelong β-blocker treatment alongside standard care was provided to all eligible Australian women (the population of patients) diagnosed with TNBC over the years 2022–2031 (the assumed lifetime of the β-blocker technology). The 10-year technology horizon was selected as a conservative estimate of the time period within which new technologies that can exceed the benefit offered by β-blockers could reasonably be developed and implemented.

## 3. Results

### 3.1. Base Case

The base case model estimated that there could be significant gains in terms of YLL, QALYs, and evLYs through the prescription of β-blockers to women with TNBC who were not already receiving this medication for another indication. The discounted model estimated 628 (95% CI 139 to 1035) YLL, 526 (116 to 865) QALYs, and 566 (125 to 932) evLYs gained in the β-blocker group compared to standard care (Table 2). The total cost of the lifetime β-blocker intervention at the population level was AUD 1,142,294 (893,676 to 1,377,890), with an overall difference in discounted health costs between β-blocker and standard care groups of AUD −935,116 (−2,365,417 to 405,350). The β-blocker intervention was dominant (cost-saving) over usual care alone in terms of both QALYs (ICER 95% CI: AUD-3284 to 1951 per QALY) and evLYs (ICER 95% CI: AUD-2757 to 783 per evLY) gained.

### 3.2. Sub-Group Analysis

Addition of a β-blocker was dominant over usual care in all age groups examined. When considering stage at diagnosis, the β-blocker intervention was dominant only in the ‘regional’ stage group (Table 3). While the intervention remained highly cost-effective in both the ‘local’ (AUD 3602/QALY) and ‘distant’ (AUD 7775/QALY) groups at the cost-effectiveness threshold of AUD 28,000, wide confidence intervals around the underlying hazard ratios for localized and distant disease introduce considerable statistical imprecision to these findings.

### 3.3. Scenario and Sensitivity Analysis

**Figure 1:**
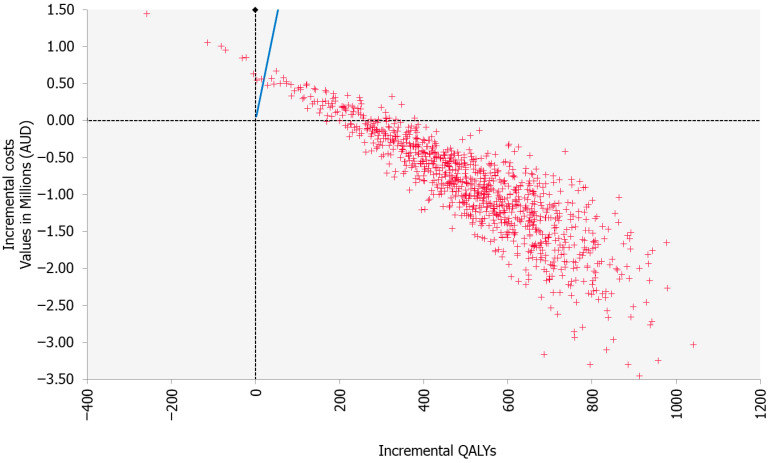
Incremental cost-effectiveness plane from probabilistic sensitivity analysis. Note: The blue line is the AUD 28,000 willingness to pay threshold. Abbreviations—AUD: Australian dollars; QALYs: quality-adjusted life years. AUD 1 = EUR 0.56.

All scenarios explored found the β-blocker intervention to be dominant over usual care, with the exception of scenario four (Carvedilol as β-blocker of choice) and scenario six (lifetime extrapolation of ongoing BC treatment costs) which both returned a highly cost-effective (although not cost-saving) ICERs of AUD 86/QALY and AUD 1229/QALY, respectively (Table 3). One-way sensitivity analysis of the base case showed that the intervention remained cost-saving over all parameter ranges examined (Supplementary Figure S2). Variation in β-blocker treatment effect on TNBC-specific survival and terminal-phase treatment costs had the greatest impact on the range of ICERs returned by the model. The probabilistic sensitivity analysis showed that the β-blocker intervention was cost-saving in 91.2% of iterations (Figure 1).

### 3.4. Value of Information Analysis Results

**Figure 2:**
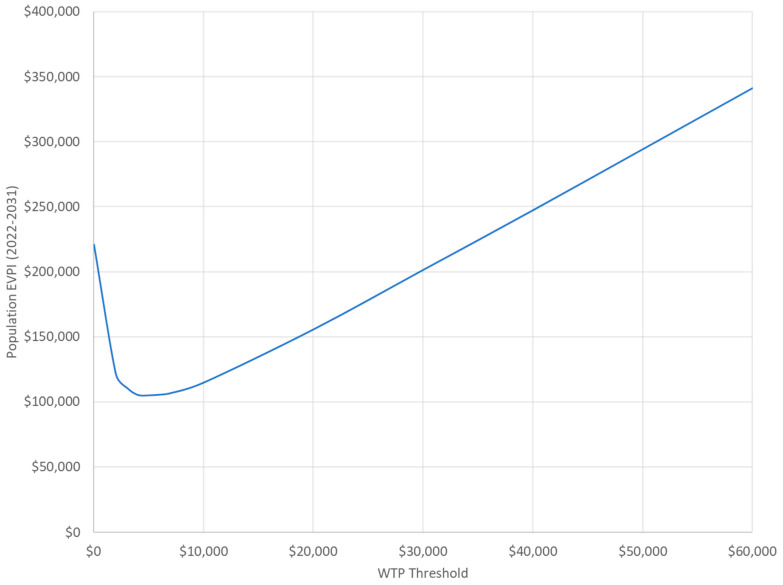
Population expected value of perfect information (EVPI) if all eligible Australian women receive treatment over years 2022–2031.

The value of information analysis estimated that the population expected value of perfect information was AUD 192,254 at the WTP threshold of AUD 28,000. This translates to approximately AUD 25 per woman commenced on β-blockers over the 10-year period examined. The population expected value of perfect information curve is provided in Figure 2.

## 4. Discussion

This preliminary cost effectiveness analysis suggests that the life-long prescription of β-blockers to women diagnosed with TNBC is cost-effective regardless of disease stage at diagnosis, and cost-saving for women diagnosed with regional cancer progression. As the incidence of this disease is low, even if implemented at a population level, the lifetime cost of β-blocker treatment for all women diagnosed with TNBC in 2022 (not already prescribed β-blockers and with no contraindication to β-blockers) is estimated to be less than AUD 1.2 million. Given the potential gains in both the quantity and quality of life years lived for this patient cohort through treatment with β-blockers alongside current standard care, and the robust safety profile of this common medication, this intervention could be considered for all women diagnosed with TNBC. This study illustrates the application of early health technology assessment to inform decisions about subsequent development, research, and/or investment in novel interventions, or, in this case, repurposing of an existing intervention for an additional clinical indication [21].

This is the first study, to our knowledge, to examine the cost-effectiveness of β-blockers as an adjunct therapy in the treatment of cancer, building on growing preclinical and clinical evidence that β-blockade slows progression of TNBC. This intervention appears to be more cost-effective than other adjunct treatments for TNBC. For example, recent studies examining the cost-effectiveness of adding atezolizumab or pembrolizumab to chemotherapy in the treatment of TNBC have returned ICERs of over USD 100,000/QALY [43,44,45,46]. The relative cost-effectiveness of β-blockers to treat TNBC reflects the opportunity that pharmaceutical repurposing can offer. In this case, the extremely low drug cost per patient is offset many times over by reductions in the end-of-life treatment costs for TNBC that are achieved through the reduction in mortality.

While economic evaluations of factors that drive cost-effectiveness are often used to support clinical development and reimbursement of drugs in early-phase clinical trials [22,47], this intervention, in contrast, seeks to evaluate the repurposing of a low-cost generic pharmaceutical that is already widely used by the adult population. Economic incentives for sponsors seeking approval for a new indication are therefore limited, which raises questions regarding who should pay for health technology assessment in the case of repurposing generic medications. The value of information analysis revealed that the value to health funders of perfect information (to eliminate uncertainty in the economic model and therefore eliminate economic risk for decision makers) is <AUD 200,000, significantly less than the estimated cost of conducting an appropriately powered phase III randomized clinical trial. Indeed, sample size calculation for a 1:1 randomized placebo-controlled trial with 80 percent power and two-tailed 0·05 significance to detect a five-year mortality difference between 12 percent (β-blockers) and 18 percent (control) reveals that a study sample of over 1100 women would be required, more than the number currently diagnosed with TNBC in Australia annually. As with all rare diseases, the challenges of recruiting the target sample for a properly powered trial would also likely increase the number of sites required, and greatly increase research costs. In the case of repurposing low-cost generic medications with a low-risk safety profile, it could be argued that a more economically appropriate approach is to implement the intervention into routine care with close monitoring of safety outcomes. A low-cost alternative, if appropriate registry data is available, is to pursue novel “target trial emulation” methodologies, where causal inference is undertaken from observational data [48].

Our focus here on TNBC reflects findings of a cohort study and meta-analysis that TNBC was the only BC molecular subtype for which β-blocker use was associated with improved BC-specific survival [16], and preclinical studies that found that β-blockade slows TNBC progression [11,12,13,14]. Possible reasons for this association may include elevated levels of sympathetic innervation of this BC subtype [49], and the neuromodulatory effects of chemotherapy drugs that form the basis of standard care for TNBC [14]. Treatment with β-blockers may more readily restore anti-cancer immunity in TNBC [17], which is associated with improved treatment response to both chemotherapy and immunotherapy [15]. An ongoing phase II non-randomized study should provide further data to support safety of β-blockers as an adjunct therapy in TNBC [50].

A key strength of this study is its use of population level data from both Australia and Norway to derive TNBC prevalence, disease staging, and mortality risk. While the treatment effect size in the base case was drawn from a single large cohort analysis of over 2000 women, we confirmed the results using a slightly different effect size derived from a published meta-analysis [16]. Comprehensive sensitivity analysis explored uncertainty around key parameters in the model. Scenario analysis was also used to explore assumptions around β-blocker drug selection, BC treatment costs, and discounting of future events.

The key limitation of this study was the unavailability of randomized controlled trial data testing the efficacy of β-blockers in improving the survival of women diagnosed with TNBC. As a consequence, treatment effect size is based on observational data alone, and the incidence of β-blocker-associated adverse events and rate of discontinuation of β-blocker therapy in women with TNBC could not be incorporated into the model. Similarly, the application of efficacy data from Norway within an Australian setting, and the application of health utility values taken from a study of younger United States breast cancer survivors, are strong assumptions of transferability, though necessary given the unavailability of Australian data for the target population. Nonetheless, findings of small phase II biomarker trials suggest that β-blockers are well tolerated in cancer patients [17,18]. Given the uncertainty of evidence supporting the risk profile of β-blockers, particularly in adults of advanced age, individual risks and benefits should be considered [51]. Women under the age of fifty were also excluded from the model and the observational data that underpins it because the current use of β-blockers in this age group is rare. However, TNBC also affects younger women, and future research should examine effectiveness and cost-effectiveness in this group.

At the population level, given that the intervention was found to be cost-saving and robust to both scenario and sensitivity analysis, cost-effectiveness is highly likely even if the true treatment effect turns out to be significantly lower than that demonstrated in the Norwegian cohort analysis. We also assumed perfect compliance with β-blocker dosing, which likely over-estimates drug use and therefore is a conservative assumption as this maximizes the cost of the intervention. As the model applied top–down, non-stage specific healthcare costs, even within the disease-stage sub-group analysis, this means that the differential ICERs are driven solely by survival differences. Furthermore, the simplicity of the model, and failure to capture the impact of the intervention on disease progression or recurrence, may have led to underestimation of potential benefits and costs that occurred later in the disease course. Care should be taken when extrapolating these findings to healthcare settings and populations that are fundamentally different to Australia.

## 5. Conclusions

The preliminary exploratory economic modelling conducted in this study indicates that the use of β-blockers as an adjunct pharmacotherapy in the treatment of TNBC is likely to be highly cost-effective, and cost-saving for the sub-group of patients that have regional tumour progression at diagnosis. Further monitoring is recommended to validate the findings of preclinical and observational analyses, and investigate the incidence, severity, and cost of β-blocker-associated adverse events in this cancer population.

## Figures and Tables

**Table 1:** Model parameters and distributions.

Variable	Fixed or Age-Specific	Distribution	Mean	Range	Source
**1-year mortality probability**					
**All-cause mortality**	Age-specific	See Supplementary Table S2.			
**TNBC-specific mortality**					
**All**	Fixed	Beta	0.039	0.034–0.041	Cancer Registry of Norway
**Localized**	Fixed	Beta	0.017	0.012–0.019	
**Regional**	Fixed	Beta	0.066	0.058–0.077	
**Distal**	Fixed	Beta	0.290	0.204–0.369	
**Effect of treatment—TNBC-specific mortality risk reduction**					
**All**	Fixed	Lognormal	0.66	0.47–0.91	Løfling et al. [16]
**Localized**	Fixed	Lognormal	0.86	0.48–1.56	
**Regional**	Fixed	Lognormal	0.54	0.34–0.86	
**Distal**	Fixed	Lognormal	0.69	0.24–2.04	
**Utilities**					
**Healthy Australian population**	Age-specific	See Supplementary Table S3.			
**Healthy Australian population average**	Fixed	Beta	0.90	0.89, 0.91	McCaffrey et al. [32]
**TNBC health state penalty**	Fixed	Beta	−0.024	−0.03, −0.02	Brown et al. [33]
**Terminal TNBC cycle penalty**	Fixed	Beta	−0.110	−0.27, 0.05	Kaur et al. [34]
**Treatment costs (AUD, 2022 prices)—β-blocker intervention**					
**Propranolol 40 mg b.d.**	Fixed	Gamma	113.95/year	9.,62, 136.29	PBS [35]
**Carvedilol 12.5 mg b.d.**	Fixed	Gamma	258.18/year	207.57, 308.78	
**Breast cancer management costs (AUD, 2022 prices)**					
**Time since diagnosis:**					
**0–1 years**	Fixed	Gamma	42,409	34,097, 50,721	Goldsbury et al. [36] (inflated to 2022 prices)
**1–2 years**	Fixed	Gamma	8745	7031, 10,459	
**2–3 years**	Fixed	Gamma	3487	2804, 4170	
**3–4 years**	Fixed	Gamma	3595	2890, 4300	
**4–5 years**	Fixed	Gamma	2839	2282, 3395	
**Terminal phase**	Fixed	Gamma	44,327	35,639, 53,015	
**Non-TNBC death**	Fixed	Gamma	5844	4698, 6989	Marquina et al. [37] (inflated to 2022 prices)

Abbreviations—AUD: Australian dollars; TNBC: triple-negative breast cancer. AUD 1 = EUR 0.56.

**Table 2:** Base case results.

	Standard Care	Standard Care Plus β-Blockers	Difference	95% CI
Total years of life lived (discounted)	6233	6870	628	139–1035
QALYs (discounted)	5166	5698	526	116–865
evLYs (discounted)	5166	5738	566	125–932
Total β-blocker treatment cost (AUD)	0	1,136,662	1,142,294	893,676–1,377,890
Total health costs (AUD—discounted)	53,029,462	52,084,447	−935,116	−2,365,417–405,350
Total population treated (n)			767	
Results per patient treated:				
Years of life lived gained			0.82	
QALYs gained			0.69	
evLYs gained			0.74	
Mean β-blocker treatment cost (AUD)			1489	
Health costs saved (AUD)			−1219	
ICER—Cost (AUD)/QALY			−1778 (Dominant)	−3284–1951
ICER—Cost (AUD)/evLY			−1653 (Dominant)	−2757–783

Abbreviations—AUD: Australian dollars; CI: confidence interval; evLYs: equal-value life years; ICER: incremental cost-effectiveness ratio; QALYs: quality-adjusted life years. AUD 1 = EUR 0.56.

**Table 3:** Results from sub-group and scenario analysis.

	Incremental YLL (Discounted)	Incremental QALYs (Discounted)	Total β-Blocker Cost (AUD)	Incremental Total Health Costs (AUD, Discounted)	ICER (AUD/QALY)
Age group:					
50–54 (n = 137)	152	129	243,184	−218,141	Dominant
55–59 (n = 162)	157	132	255,397	−194,415	Dominant
60–64 (n = 158)	125	104	213,306	−182,693	Dominant
65–69 (n = 170)	127	105	188,022	−179,220	Dominant
70–74 (n = 189)	92	75	158,525	−159,948	Dominant
75–79 (n = 143)	33	28	78,229	−63,932	Dominant
Tumour stage at diagnosis:					
Local (60.5%)	82	68	773,279	246,207	3602
Regional (35.6%)	427	357	365,685	−604,284	Dominant
Distal (3.9%)	29	24	13,740	190,226	7775
Scenarios:					
(1) Discount rate 0%	1179	978	1,136,662	−1,338,484	Dominant
(2) Discount rate 3.5%	754	629	1,136,662	−1,049,370	Dominant
(3) Alternative RR of BC-related death (0.74)	478	400	1,102,710	−503,655	Dominant
(4) Carvedilol as drug of choice	636	533	2,575,269	45749	86
(5) Increase propranolol dose to 80 mg twice daily	636	533	2,273,324	−162,199	Dominant
(6) Including lifetime BC management costs	636	533	1,136,662	654,216	1229

Abbreviations—AUD: Australian dollars; BC: breast cancer; ICER: incremental cost-effectiveness ratio; RR: risk ratio; QALYs: quality-adjusted life years; YLL: years of life lived. AUD 1 = EUR 0.56.

## Data Availability

All data used in modelling is publicly available as referenced throughout the manuscript. Where published data was consolidated, modified, or combined through analysis this is summarized in the Methods or Supplementary Resources.

## Supplementary Materials

The following supporting information can be downloaded at: https://www.mdpi.com/article/10.3390/healthcare13222929/s1, Figure S1: Markov model showing possible health states; Figure S2: Tornado diagram showing results of one-way sensitivity analysis on the ICER; Table S1: Projected breast cancer incidence in Australian women (2022); Table S2: Age-specific all-cause mortality rate and survival function for Australian females (2019); Table S3: Utility norms for Australian female population used in model.

## Abbreviations

The following abbreviations are used in this manuscript:

AUD	Australian dollar
BC	Breast cancer
evLY	Equal-value life year
HTA	Health technology assessment
ICER	Incremental cost-effectiveness ratio
QALY	Quality-adjusted life year
TNBC	Triple-negative breast cancer
YLL	Years of life lived

## Appendix A

Model validation checklist—Adjunct Beta-blockers for triple-negative breast cancer.

**Category**	**Item**	**Checked**
Logical tests	Set all utility values equal to 1. QALYs should equal LYs.	Yes
	Set all utility values to zero. There should be zero QALYs accrued for all included treatments.	Yes
	Set all mortality rates (including background mortality) to 1. All patients should be dead in cycle 1, but still produce (some) expected costs and QALYs (due to half cycle, and one-off costs/disutilities).	Yes
	Set mortality rate (including background mortality) at age X to 1. All patients should survive until age X, and still produce expected costs and QALYs.	Yes
	If included, set all AE probabilities to zero. Make sure that no AEs occur, and that AE-related costs and disutilities are also estimated to be zero.	Yes
	Set unit costs for all included treatments to zero. Estimated treatment costs should be zero.	Yes
	Halve and double treatment unit costs for each treatment. Estimated undiscounted treatment costs should also halve and double in response.	Yes
	For other included cost categories: halve, double, and set to zero. Ensure that all undiscounted model results respond as expected.	Yes
	If included as an input: increase and decrease treatment durations. Do treatment costs increase and decrease appropriately in response?	N/A
	Explore higher and lower time horizons. LYs, QALYs and total costs should increase/decrease with longer/shorter time horizons. Set time horizon to zero—all costs and outcomes should be zero/undefined.	N/A
	Set the discount rate of benefits to 100%. Total QALYs should dramatically decrease. Repeat for cost discount rate.	Yes
	Set the discount rate of benefits to 0%. Total discounted QALYs should increase and match undiscounted results exactly. Repeat for cost discount rate.	Yes
Technical implementation	Check that half cycle correction has been appropriately applied.	N/A
	Check that background mortality is correctly applied (for the correct age, adjusted for cycle length, reactive to changes in age and gender distribution). Pay special attention to the last model cycle, and any assumptions made for ages that fall outside of the life table (e.g., 100+).	Yes
	Has discounting been appropriately applied using the correct formula? ((1 + p)^(−t)). And is it implemented separately for costs and benefits? (Check cell references).	Yes
	Check that the sum of all health state membership in the model sums to 1 for all cycles. Check that this is not simply the result of one state’s membership being equal to 1 minus all others (this can hide errors).	Yes
	Check that probabilities and rates have been handled correctly (i.e., rates are converted to probabilities before being used as transitions).	Yes
	Check that the starting distribution of health states is correct, and consistent across the included treatments. Check that it makes sense given the decision problem (e.g., patients who have the disease, vs. patients who have been diagnosed with disease).	Yes
	Ensure that the cumulative probability to die in any given cycle is equal to or greater than that of the age-matched general population mortality. Use the background mortality tables to check.	Yes
	Confirm that the relationship with the cycle length and time horizon is correct.	Yes
	Following on from the above, ensure that treatment durations are correct by confirming that the model is incurring treatment costs for the correct number of cycles. Ensure any stopping rules are appropriately timed.	Yes
	Confirm that disutilities are correctly subtracted from QALYs, by ensuring that duration is taken into account in the calculations.	Yes
