# Supplementary material for: Preliminary Cost-Effectiveness of Re-Purposing β-Blockers as an Adjunct Treatment for Women with Triple-Negative Breast Cancer
Source: Healthcare (Basel). 2025 Nov 15;13(22):2929. doi: 10.3390/healthcare13222929 (PMC12652519; doi:10.3390/healthcare13222929)
Supplement: Supplementary file 1 [file healthcare-13-02929-s001.zip › healthcare-3911652-supplementary.pdf]

Online Resources

Preliminary cost-effectiveness of re-purposing β-blockers as an adjunct treatment for women with triple negative breast cancer

Contents

Supplementary Table S1. Projected breast cancer incidence in Australian women (2022) 2

Supplementary Table S2. Age-specific all-cause mortality rate and survival function for Australian females (2019) 3

Supplementary Table S3. Utility norms for Australian female population used in model. 4

Supplementary Figure S1. Markov model showing possible health states 5

Supplementary Figure S2. Tornado diagram showing results of one-way sensitivity analysis on the ICER 6

References 7

Supplementary Table S1. Projected breast cancer incidence in Australian women (2022)

Age group	BC incidence rate (per person year) [a]	Projected BC cases diagnosed in Australia [a]	% BC cases classified as TNBC sub-type [b]	TNBC cases receiving intervention [c]
**50-54**	0.0027	1976	8.1	109
**55-59**	0.0027	2013	9.3	130
**60-64**	0.0034	2026	7.4	127
**65-69**	0.0041	1977	7.6	136
**70-74**	0.0044	1988	9.3	151
**75-79**	0.0040	2047	9.8	114
**Total**		12026		767

[a] Incidence derived from published data: Australian Institute of Health and Welfare (3), Australian Bureau of Statistics population projections by age (2022) (2)
[b] Proportion of BC that are TNBC molecular sub-type derived from the Norwegian Cancer Biobank.
[c] Excludes the 14.8% of women with BC already prescribed β-blockers for a comorbid indication (3), and 20% of women assumed to have a contraindication to β-blocker use.

Abbreviations – BC: breast cancer; TNBC: triple negative breast cancer.

Supplementary Table S2. Age-specific all-cause mortality rate and survival function for Australian females (2019)

Age	Mortality rate	Survival function
50	0.0019	0.9981
51	0.0021	0.9979
52	0.0023	0.9977
53	0.0026	0.9974
54	0.0028	0.9972
55	0.0031	0.9969
56	0.0035	0.9965
57	0.0038	0.9962
58	0.0042	0.9958
59	0.0046	0.9954
60	0.0051	0.9949
61	0.0056	0.9944
62	0.0062	0.9938
63	0.0069	0.9931
64	0.0076	0.9924
65	0.0084	0.9916
66	0.0092	0.9908
67	0.0102	0.9898
68	0.0112	0.9888
69	0.0124	0.9876
70	0.0137	0.9863
71	0.0151	0.9849
72	0.0167	0.9833
73	0.0184	0.9816
74	0.0203	0.9797
75	0.0224	0.9776
76	0.0247	0.9753
77	0.0273	0.9727
78	0.0301	0.9699
79	0.0332	0.9668
80	0.0366	0.9634
81	0.0404	0.9596
82	0.0446	0.9554
83	0.0492	0.9508
84	0.0543	0.9457
85	0.0599	0.9401

Notes: Rate per individual year of age extrapolated from AIHW GRIM dataset(4) using a polynomial function for line of best fit:

Mortality rate = 0.0000000515*age^3 - 0.0000030107*age^2 + 0.0000695603*age - 0.0002528937

Supplementary Table S3. Utility norms for Australian female population used in model.

Age	Mean	SD
**50**	0.87	0.16
**51**	0.87	0.16
**52**	0.87	0.16
**53**	0.87	0.16
**54**	0.87	0.16
**55**	0.88	0.15
**56**	0.88	0.15
**57**	0.88	0.15
**58**	0.88	0.15
**59**	0.88	0.15
**60**	0.88	0.15
**61**	0.88	0.15
**62**	0.88	0.15
**63**	0.88	0.15
**64**	0.88	0.15
**65**	0.87	0.16
**66**	0.87	0.16
**67**	0.87	0.16
**68**	0.87	0.16
**69**	0.87	0.16
**70**	0.87	0.16
**71**	0.87	0.16
**72**	0.87	0.16
**73**	0.87	0.16
**74**	0.87	0.16
**75**	0.82	0.15
**76**	0.82	0.15
**77**	0.82	0.15
**78**	0.82	0.15
**79**	0.82	0.15
**80**	0.82	0.15
**81**	0.82	0.15
**82**	0.82	0.15
**83**	0.82	0.15
**84**	0.82	0.15
**85**	0.82	0.15

Source: McCaffrey et al. 2016.(5)

Supplementary Figure S1. Markov model showing possible health states

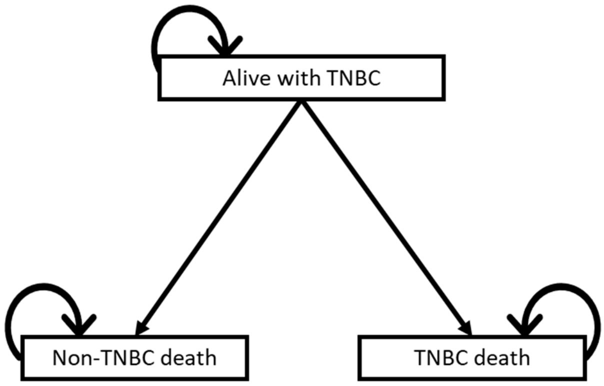

Abbreviations. TNBC: Triple-negative breast cancer.

Supplementary Figure S2. Tornado diagram showing results of one-way sensitivity analysis on the ICER

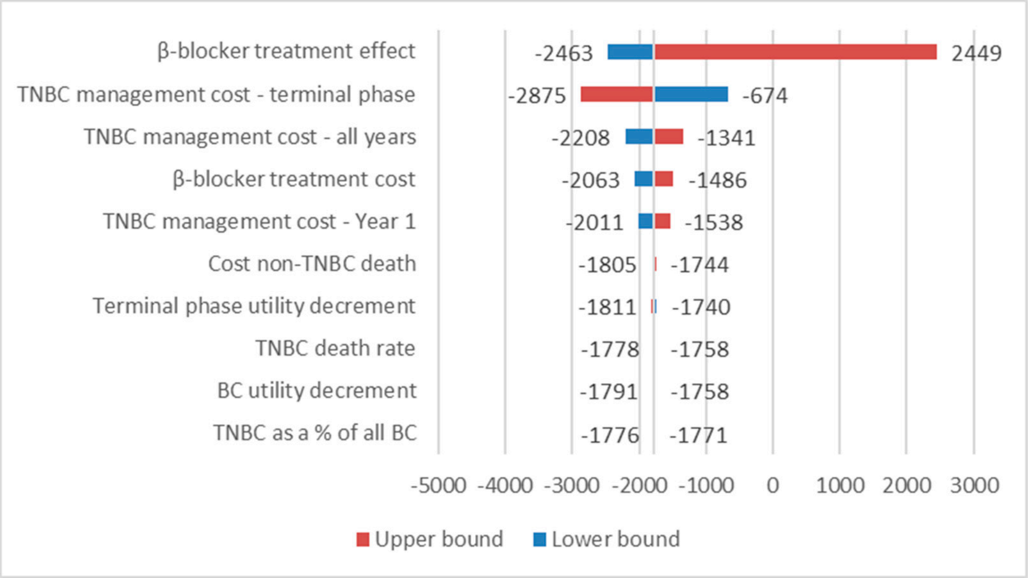

Abbreviations – BC: breast cancer; ICER: incremental cost effectiveness ratio; TNBC: triple negative breast cancer. All costs are shown in Australian dollars.

Note: A Tornado Diagram is a graphical tool used in cost-effectiveness analysis (CEA) to display the results of a one-way sensitivity analysis. It illustrates how application of the upper and lower confidence range limit of each individual model parameter (as listed in Table 1) influences the incremental cost-effectiveness ratio. For example, when the lower bound of the 95% confidence interval for the β-blocker treatment effect (hazard ratio = 0.47) is applied instead of the mean (0.66), the ICER returned by the model will be AUD-2463 per QALY gained (cost-saving). However, if the upper bound of the confidence interval is applied (0.91), the intervention will be cost-effective but no longer be cost-saving (ICER = AUD2449/QALY). The β-blocker treatment effect was the most influential parameter applied in the model in terms of the impact of uncertainty.

References

1. Australian Institute of Health and Welfare. Cancer data in Australia 2021 – Web Report. Accessed 1 December 2023. Available from: https://www.aihw.gov.au/reports/cancer/cancer-in-australia-2021/data.

2. Australian Bureau of Statistics. Australian Population Clock and Pyramid 2023 Accessed 1 December 2023. Available from: https://www.abs.gov.au/statistics/people/population/population-clock-pyramid.

3. Løfling LL, Støer NC, Sloan EK, Chang A, Gandini S, Ursin G, et al. β-blockers and breast cancer survival by molecular subtypes: a population-based cohort study and meta-analysis. British Journal of Cancer. 2022;127(6):1086-96.

4. Australian Institute of Health and Welfare. General Record of Incidence of Mortality (GRIM) data. Accessed 11 July 2023. Available from: https://www.aihw.gov.au/reports/life-expectancy-deaths/grim-books/contents/general-record-of-incidence-of-mortality-grim-data.

5. McCaffrey N, Kaambwa B, Currow DC, Ratcliffe J. Health-related quality of life measured using the EQ-5D–5L: South Australian population norms. Health and Quality of Life Outcomes. 2016;14(1):1-12.
